# Changes in Maximal Strength and Home Run Performance in NCAA Division I Baseball Players Across 3 Competitive Seasons: A Descriptive Study

**DOI:** 10.3390/jfmk6010004

**Published:** 2021-01-02

**Authors:** W. Guy Hornsby, Abigail L. Tice, Jason D. Stone, Justin J. Merrigan, Joshua Hagen, John P. Wagle, Aaron J. Cunanan, Michael H. Stone

**Affiliations:** 1 College of Physical Activity and Sport Sciences, West Virginia University, Morgantown, WV 26505, USA; 2 Rockefeller Neuroscience Institute, West Virginia University, Morgantown, WV 26505, USA; jason.stone1@hsc.wvu.edu (J.D.S.); justin.merrigan@hsc.wvu.edu (J.J.M.); joshua.hagen@hsc.wvu.edu (J.H.); 3 Department of Nutrition, Food and Exercise Science, Florida State University, Tallahassee, FL 32306, USA; at19r@my.fsu.edu; 4 Kansas City Royals, Kansas City, MO 64129, USA; johnwagle9@gmail.com; 5 San Francisco Giants, San Francisco, CA 94107, USA; aaron.cunanan@gmail.com; 6 Department of Sport, Exercise, Recreation, and Kinesiology, East Tennessee University, Johnson City, TN 37604, USA; STONEM@mail.etsu.edu

**Keywords:** baseball, peak force, rate of force development, isometric strength, home runs

## Abstract

The purpose of this longitudinal, descriptive study was to observe changes in maximal strength measured via isometric clean grip mid-thigh pull and home runs (total and home runs per game) across three years of training and three competitive seasons for four National Collegiate Athletic Association (NCAA) Division 1 baseball players. A one-way repeated measures analysis of variance (ANOVA) was performed, revealing significant univariate effects of time for peak force (PF) (*p* = 0.003) and peak force allometrically scaled (PFa) (*p* = 0.002). Increases in PF were noted from season 1 to season 2 (*p* = 0.031) and season 3 (*p* = 0.004), but season 2 was not significantly different than season 3 (*p* = 0.232). Additionally, increases in PFa were noted from season 1 to season 2 (*p* = 0.010) and season 3 (*p* < 0.001), but season 2 was not significantly different than season 3 (*p* = 0.052). Home runs per game rose from the 2009 (0.32) to 2010 season (1.35) and dropped during the 2011 season (1.07). A unique aspect of the study involves 2010 being the season in which ball-bat coefficient of restitution (BBCOR) bats were introduced to the NCAA competition.

## 1. Introduction

Resistance training (RT) enhances muscle strength and size through various applications, with important implications across a wide spectrum of athletic populations, including baseball players [1]. A 2005 [2] survey study of head major league baseball strength coaches demonstrated that most respondents (21 of 30) employ a periodized strength program (18 of 21). The “drug era” of the 1990s established a shift in many people’s perceptions of the importance of strength and its development in baseball, particularly its effect on power hitting (e.g., home runs, slugging % and extra base hits) [3]. Strength has been associated with higher hitting related variables [4,5,6], typically in controlled field-based testing or laboratory-based testing (e.g., bat speed, batted ball velocity).

For hitters, it may therefore be logical to engage in an RT program to capitalize on these downstream effects on swing and batted ball velocity [4,7,8]. Increased bat swing velocity provides hitters with a greater decision time interval, allowing the ball to be closer to them before initiating their swing [4,7]. Additionally, increasing bat swing velocity can increase the speed at which the ball is projected off of the bat (batted ball velocity), increasing the chances of a productive outcome [4,7]. Various training protocols have been shown to increase bat swing velocity, including the use of free weights, bats of various weights, plyometric exercises, medicine balls, and weightlifting movements [5,6,7,9]. However, though strength and hitting ability have a well-established relationship [10,11,12,13], it is uncertain whether long-term changes (e.g., years) in strength coincide with improvements in “on-field” hitting outcomes, such as home runs. An investigation on professional baseball players throughout an entire organization (rookie ball through Major League) displayed that speed, agility, and lower body power were the strongest predictors of baseball-specific performance [10]. These force-related qualities of speed, agility and lower body power have been shown to be improved through increases in maximal and relative strength [1]. Therefore, having baseball hitters participate in a RT program likely influences their overall development.

Many challenges exist when trying “connect” on field data, such as in-game hitting statistics, with lab measures. Specifically, for baseball, on-field data can dramatically reduce the sample size (e.g., approximately 25 to ≤ 9) and long-term data can be difficult to obtain as it requires consistent testing protocols over several years. For example, for a collegiate roster of 30 to 35 student athletes, roughly 15–18 may be pitchers that do not hit, leaving only 12–15 for a potential sample. Of those hitters, there may only be 7–8 that are everyday players and it is highly unlikely that those 7–8 are the same players for multiple seasons in a row. Despite these challenges, sport scientists have discussed that the observation of athletes in real world settings over extended periods of time has an important place within the wide spectrum of sport science work [14]. The purpose of the present, descriptive study was to observe changes in maximal strength alongside changes in home runs (the most productive batted ball) during three collegiate baseball seasons. A unique aspect of this study is that four players remained in the starting lineup for three straight collegiate baseball seasons.

## 2. Methods

During the 2009, 2010, and 2011 competitive seasons, four National Collegiate Athletic Association (NCAA) Division I starters (1 freshman and 3 sophomores, 86.0 ± 10.1 kg body mass, 184.9 ± 2.2 cm height, 76.8 ± 9.7 kg lean body mass, 12.1 ± 1.8 body fat percentage) with a minimum of 100 at bats (the required inclusion criteria), each of three seasons, were selected to take part in an athlete monitoring program. Specifically, body composition, strength assessment and home runs were tracked across the 3 seasons (2009, 2010, and 2011). The two primary laboratory measures included in the athlete monitoring program were 1) body composition assessment via air displacement plethysmography and 2) isometric mid-thigh pull testing, which allowed for the assessment of strength related characteristics. Home run data were acquired from publicly available NCAA statistics (http://www.ncaa.org/championships/statistics/baseball-statistics). The study protocol was approved by the East Tennessee State University Institutional Review Board.

## 3. Testing Procedures

**Figure 1:**
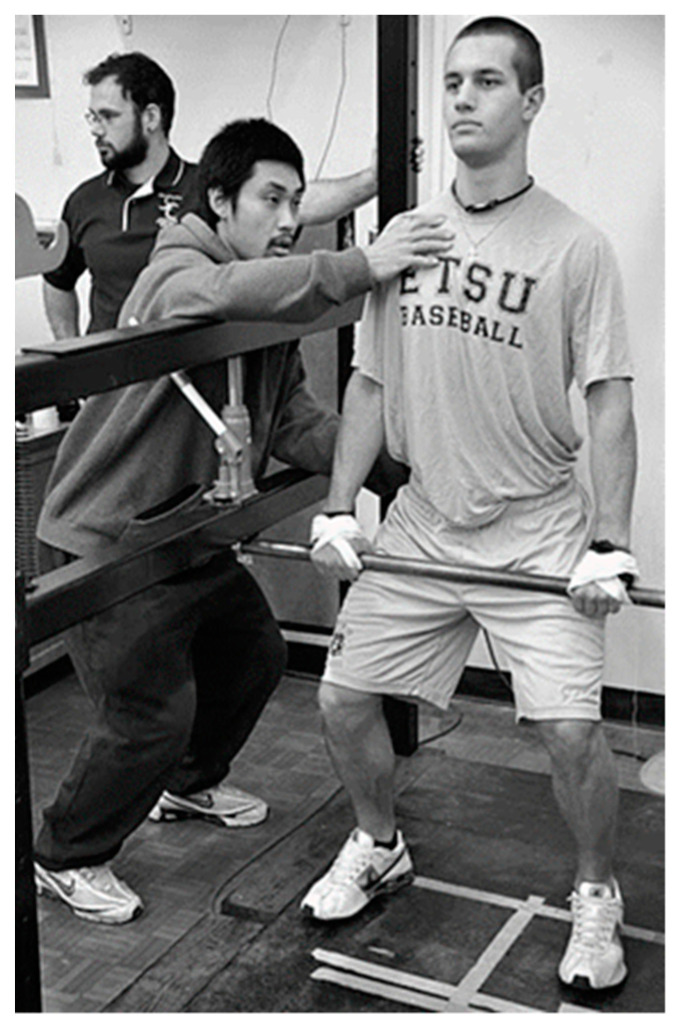
An investigator placing subject in proper clean grip mid-thigh pull position.

Height (measured without shoes) and body mass were recorded to the nearest 0.01 cm and 0.02 kg, respectively, using a stadiometer (Detecto, Webb City, MO, USA) and digital scale (BOD POD; Cosmed USA, Chicago, IL, USA). Air displacement plethysmography (BOD POD; Cosmed USA, Chicago, IL, USA) was used to measure body composition (fat and fat free mass) via previously established and validated methods [15,16]. Isometric mid-thigh pull (IMTP) testing [17] was performed on a 91.4 × 91.4 cm force plate (frequency = 1000 hz, 2nd order Butterworth low-pass filter at 10 Hz) (Rice Lake Weighting Systems, Rice Lake, WI) inside a custom designed power rack that allows the fixation of the bar to fit athletes of various heights. Commercially available software was used for subsequent IMTP analyses (ForceDecks, London, UK). The testing of both body composition and IMTP was performed on three different dates (16/10/2008, 29/1/2010 and 1/2/2011) each corresponding to a different season (season 1, 2 or 3). Knee angle was measured using a hand-held goniometer to verify knee angle consistency between 125°–135° and a stature and stance mimicking the power position used to initiate the 2nd pull of a clean (Figure 1) [17]. Bar height was based on this desired position [17]. Subjects were permitted to exert minimal tension before testing began to ensure no slack in the subject’s posture prior to exertion. The subject’s body position stabilization was verified by observation and force trace before testing began. Upon onset of testing, the athlete was told to pull as fast and hard as possible and given a countdown before each isometric mid-thigh pull (“3, 2, 1, pull!”). Two warmups were performed, one at 50% and one at 75% of the athlete’s perceived maximal exertion before the test attempt commenced [18]. Strong verbal encouragement was given to the athlete as they performed the 2 maximal effort trials. The two test attempts were performed at 100% of the athlete’s maximal exertion and the mean of the two trials was recorded for both peak force (PF) and peak force allometrically scaled (PFa) (PF/bdm 0.67) [19]. Athletes hands were strapped and then subsequently taped to the bar to ensure that they were not limited by grip strength [17].

## 4. Statistics

The normality of data was checked and confirmed via a visual inspection of Q-Q plots and Shapiro–Wilk tests. A one-way repeated measures analysis of variance (ANOVA) was performed for each dependent variable (body mass, body fat percentage, PF, PFa) over time (year 1, 2, 3). If a significant univariate effect was noted, follow-up pairwise t-test comparisons were conducted using a Bonferroni correction. Additionally, Cohen’s d effect sizes and their 95% confidence intervals were calculated. Significance was defined as *p* ≤ 0.05. Percentage changes were calculated between subsequent seasons for home runs. All statistical procedures were conducted using R, version 3.6.2 (R Core Team, Vienna, Austria; https://www.R-project.org).

## 5. Results

**Figure 2:**
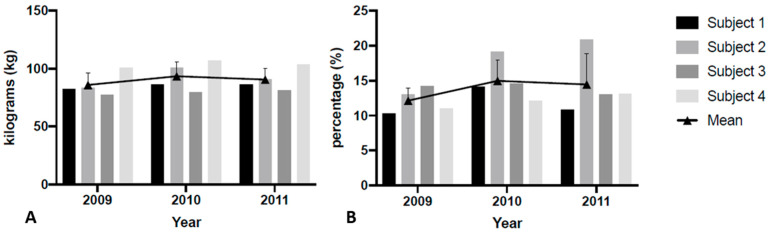
Body mass (**A**) and body fat percentage (**B**) changes over the course of three seasons.

**Figure 3:**
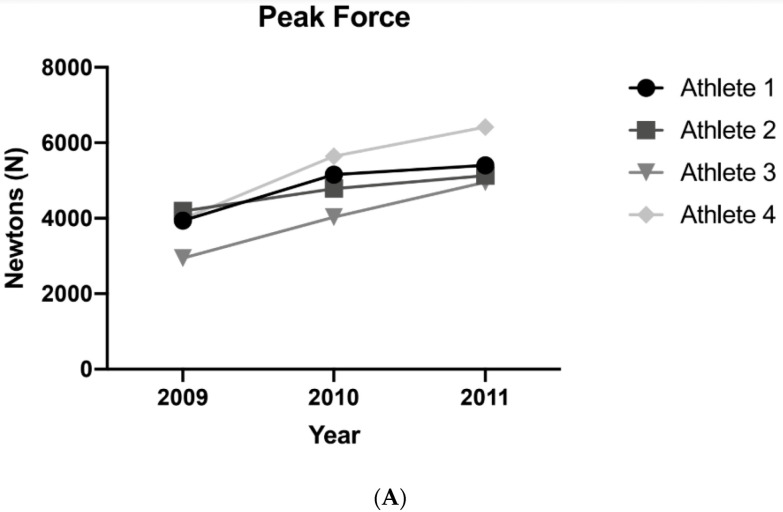

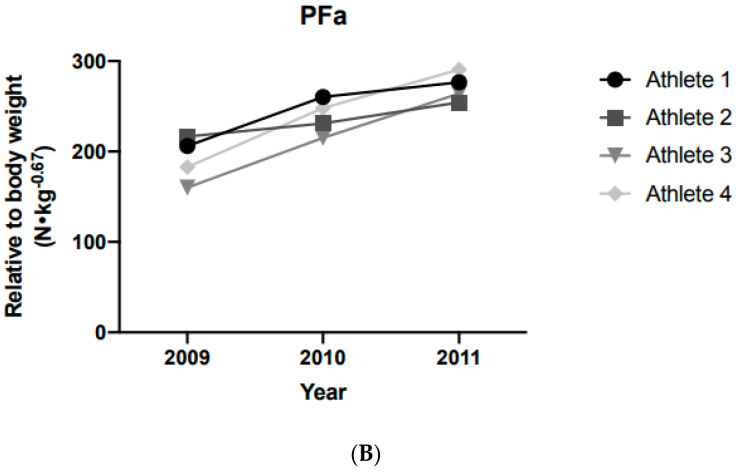
Changes in absolute (**A**) isometric peak force and (**B**) allometrically scaled peak force over 3 seasons.

**Figure 4:**
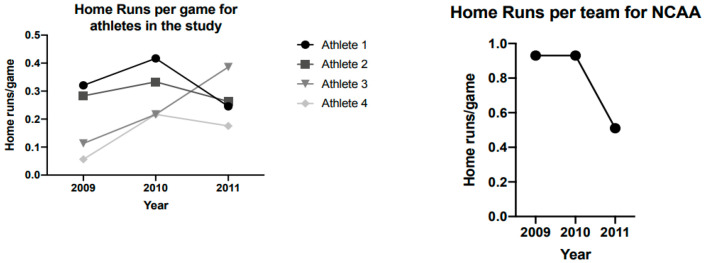
Average home runs per game in National Collegiate Athletic Association (NCAA) Division I Baseball compared to average home runs per game for the four athletes in the study for three consecutive seasons.

Body mass (*p* = 0.560; Figure 2A) and body fat percentage (*p* = 0.328; Figure 2B) remained unchanged over the course of the three seasons. There were significant univariate effects of time for PF (*p* = 0.003) and PFa (*p* = 0.002). Increases in PF were noted from season 1 to season 2 and season 3, but season 2 was not significantly different than season 3 (Table 1). Additionally, increases in PFa were noted from season 1 to season 2 and season 3, but season 2 was not significantly different than season 3 (Table 1, Figure 3B). Table 2 displays home run variations across the seasons and demonstrates that home runs increased from 2009 to 2010 season and all but one subject experienced a decrease in home runs from 2010–2011. Figure 3 illustrates changes in PF and Pfa, while Figure 4 illustrates changes in home runs for the 4 subjects compared to the rest of NCAA Division 1. 

## 6. Discussion

The main findings of this observational study were (1) players experienced increases in PF and PFa across all three years, while (2) home runs and home runs per game increased from 2009 (41 and 0.32) to 2010 (81 and 1.035), but decreased from 2010 to 2011 (61 and 1.07). Hitting home runs is certainly a multifactorial endeavor, however it is generally accepted that increased strength can be helpful in improving home run hitting ability. The improvements in strength experienced by the subjects may have contributed to hitting performance by increasing bat swing velocity and batted-ball velocity, [7] however, these aspects were not measured and are purely speculative. Percent difference changes for each athlete for each testing measurement demonstrated that each of the four athletes responded similarly (i.e., increased) for PF and PFa.

An interesting aspect of the current study was that for the 2011 season (season three), the NCAA instituted a change from aluminum to ball-bat coefficient of restitution (BBCOR) bats in an effort to decrease the batted-ball velocity as a means of reducing the risk of serious injury to pitchers and other field players [20]. The coefficient of restitution is a commonly used measure to assess the “bounce” of a given baseball bat, NCAA BBCOR bats must not exceed 0.05 (NCAA) [20]. In 2011, average home runs per season in NCAA Division I baseball (*n* = 292 teams) dropped from 65.2 home runs in 2010 to 28.8 home runs in 2011. This 77% drop between seasons can be compared to each of these four players individual percent changes in home runs between these two seasons (−33.3%, 7.14%, −30%, and −13.6%). It is reasonable to assume that this drop off can be at least partially attributed due to a reduced total distance of ball flight via alterations to bat composition. Thus, stronger athletes may have an advantage in handling the switch to a bat with a reduced trampoline effect, a “spring like” effect some aluminum bats have that allows the bat to “spring back” to shape after being struck. Notable is that the relative drop in home runs per game for the four subjects was less (−28%) than the national average (−44%) (Figure 4). Indeed, the drop in home runs in 2011 across the country was dramatic as home run numbers descended to almost pre-aluminum bat values, the lowest number of home runs per game since 1974 (http://fs.ncaa.org/Docs/stats/baseball_RB/reports/TrendsYBY.pdf). An interesting home run comparison for the four players is the first season (2009) compared to the third and last season (2011); with multiple years of strength development, the four subjects hit more home runs (41 vs. 61) with the less responsive BBCOR bats compared to the pre 2011 aluminum bats. Certainly, becoming more accustomed to collegiate level pitching and many other factors also likely contributed. Over the three years of the study, the team that the four subjects played for steadily rose within the NCAA national home run statistics, rising from 39th (2009) to 12th (2010) to 1st (2011) in home runs per game. During this rise in home runs, nationally the four subjects contributed substantially to the team’s overall home run output; 89% in 2009, 87% in 2010 and 71% in 2011. In 2011, the four subjects alone (as a team unto themselves), with their 61 home runs would have finished tied for eighth in total home runs (and four home runs away from fifth place).

It has been suggested that perhaps, due to the challenging nature of the collegiate season, (i.e., February to June followed by 2–3 months of summer baseball) that after a year being in a collegiate program, the focus of the weight room should shift from strength enhancement to strength maintenance and power development [21]. Based on these data, baseball players can continue to increase strength across multiple years within a collegiate strength and conditioning program. Hitting and home run performance is multifaceted (e.g., technique, the opponent pitching, etc.); however, strength is certainly a factor in hitting performance [1,10]. Increases in strength may not only improve home run capabilities, but also help hitters using the less responsive BBCOR bats to produce home runs.

It is well established that long-term RT leads to increases in strength and that increases can be beneficial for baseball (and general sport) performance [2,8]. The results of this study suggest a potential influence of strength on hitting performance. Indeed, previous studies have found improvements in several hitting-related factors (i.e., bat swing velocity and batted ball velocity) following RT [5,6,9]. The collective evidence suggests that strength training may be beneficial for hitting performance among collegiate baseball players. Additionally, studies investigating the isometric strength capabilities of baseball players [22,23] and athletes of various sports, the subject’s third year, mean that PFa of 271 N·kg^−0.67^ could very well be considered “strong” [24,25]. Admittedly, more data on baseball players are necessary to build normative data to allow for classifications and recommendations.

Lastly, while “real world” descriptive studies that follow athletes over extended periods of time can be incredibly valuable, these studies are not without limitations. Limitations are specifically related to the inability to generalize these results beyond the sample. An additional limitation is that season 1’s testing occurred during the middle of the fall semester, while seasons 2 and 3 occurred right before the start of spring practice. Ideally testing dates would remain consistent from one year to the next.

## 7. Practical Application

Hitting and home run performance are certainly multifaceted endeavors (e.g., technique, the opponent pitching, etc.). However, the magnitude of force production is a well-established factor in hitting performance [1,4,7]. This study did not consider the specific dynamics of the games nor did it involve tracking the specifics of the training program. Future work should involve close monitoring of the athletes’ long-term training along with strength alterations and baseball performance. Based on these data, baseball players can continue to increase strength across multiple years within a collegiate strength and conditioning program. Increases in strength may aid in the ability to hit home runs.

## Figures and Tables

**Table 1:** Effect size and 95% confidence interval for changes across seasons.

	Season 1 to 2	Season 1 to 3	Season 2 to 3
Body Mass	0.65 (−0.85, 1.97)	0.46 (−1.00, 1.80)	−0.25 (−1.61, 1.17)
Body Fat Percentage	1.17 (−0.46, 2.48)	0.69 (−0.82, 2.01)	−0.14 (−1.51, 1.27)
Isometric Peak Force	1.83 (0.00, 3.18)	2.82 (0.62, 4.28) *	0.86 (−0.69, 2.18)
Allometrically Scaled Peak Force	2.09 (0.17, 3.46)	3.81 (1.18, 5.44) *	1.83 (0.00, 3.18)

For effect size, d = 0–0.2; small, d = 0.2–0.6; moderate, d = 0.6–1.2; large, d = 1.2–2.0; and very large d > 2.0. For 95% confidence interval * denotes statistical significance.

**Table 2:** Home runs per season.

Player	Season	Home Runs	Percent Change (%) between Seasons
Subject 1	2009	15	
	2010	20	33.3
	2011	15	−25.0
Subject 2	2009	6	
	2010	13	117
	2011	14	7.69
Subject 3	2009	3	
	2010	13	333
	2011	10	−23.1
Subject 4	2009	17	
	2010	25	47.1
	2011	22	−12.0

## Data Availability

All data is available within the manuscript.
